# A Hyaluronan Hydrogel Scaffold for Culture of Human Oral Mucosal Epithelial Cells in Limbal Stem-Cell Therapy

**DOI:** 10.3390/bioengineering6040097

**Published:** 2019-10-23

**Authors:** Mazyar Yazdani, Aboulghassem Shahdadfar, Catherine Joan Jackson, Tor Paaske Utheim

**Affiliations:** 1 Department of Medical Biochemistry, Oslo University Hospital, Ullevål, 0450 Oslo, Norway; catherinejoanjackson@gmail.com (C.J.J.); utheim2@gmail.com (T.P.U.); 2 Center for Eye Research, Department of Ophthalmology, Oslo University Hospital, Ullevål, 0450 Oslo, Norway; aboulghassem.shahdadfar@medisin.uio.no; 3 The Norwegian Dry Eye Clinic, 0366 Oslo, Norway; 4 Institute of Oral Biology, Faculty of Dentistry, University of Oslo, 0318 Oslo, Norway; 5 Department of Plastic and Reconstructive Surgery, Oslo University Hospital, 0450 Oslo, Norway; 6 Department of Ophthalmology, Stavanger University Hospital, 4011 Stavanger, Norway; 7 Department of Ophthalmology, Sørlandet Hospital Arendal, 4604 Arendal, Norway

**Keywords:** limbal stem cell deficiency, hyaluronan hydrogel scaffold, human oral mucosal epithelial cells (OMECs), stem cell-based therapy, transplantation

## Abstract

Hyaluronan (HA), a major component of the extracellular matrix, plays a key role in cell proliferation, growth, survival, polarization and differentiation. We investigated the optimization of a HA hydrogel scaffold for culture of human oral mucosal epithelial cells (OMECs) for potential application in limbal stem cell therapy. The effect of the optimized scaffold on OMEC cell sheet morphology, cell metabolic activity and expression of genes associated with stemness, adherence and cell damage was studied. The results indicate that HA hydrogels crosslinked with polyethylene glycol diacrylate (PEGDA) failed to support OMEC attachment and growth. However, HA hydrogel scaffolds dried for three days and coated with 1 mg/mL collagen IV produced a full OMEC sheet. Cell morphology was comparable to control after three weeks culture, maintaining 76% metabolic activity. Of apoptosis-related genes, the pro-apoptotic markers *CASP3* and *BAX2* were upregulated and downregulated, respectively, compared to control whereas the anti-apoptotic marker *BCL2* was downregulated. The expression level of stemness genes *ΔNp63α* and *ABCG2* was significantly higher than control. Genes associated with improved scar-less wound healing (integrin-αV) and protection of the ocular surface (cadherin-1) had ~3-fold increased expression. These data suggest that our optimized HA-hydrogel scaffold could enhance culture of OMEC cell sheets for use in ocular reconstruction.

## 1. Introduction

Limbal stem-cell deficiency (LSCD) is a disease resulting from injury or loss of the LSC pool caused by cellular damage and/or changes in the cellular microenvironment [1,2]. It can be treated by several methods. Ex vivo expansion of limbal epithelial cells (LECs) from the patient’s healthy eye has been used for treatment of unilateral LSCD [3]. As bilateral LSCD is more prevalent than unilateral disease, there is increased interest in exploring alternative autologous cell sources for ocular surface reconstruction. The first alternative cell type used was oral mucosal epithelial cells (OMECs) from rabbits [4] and humans [5]. Later, other cell types such as embryonic stem cells [6], conjunctival epithelial cells [7], bone marrow-derived mesenchymal stem cells [8], epidermal adult stem cells [9], immature dental pulp stem cells [10], follicle bulge-derived stem cells [11] and umbilical cord-lining stem cells [12] were introduced. Cell sheets are typically cultured on a carrier scaffold for transplantation to the ocular surface [13]. 

Choice of carrier scaffold is crucial to prevent differentiation and loss of stem-cell phenotype. The phenotypic state is an important indicator of healthy cells and it is known that the success rate of regeneration in damaged tissue depends on the degree of stemness in the transplant sheet rather than the number of cells [14]. Several natural and synthetic polymer materials have been developed for use as scaffolds such as human amniotic membrane, fibrin, siloxane hydrogel contact lenses, human anterior lens capsule, collagen, plastic compressed collagen, crosslinked collagen, electrospun and magnetically oriented scaffolds [15]. Several studies suggest hyaluronan (HA) hydrogel scaffolds as a potential carrier scaffold for use in treatment of LSCD. HA hydrogel scaffolds chemically cross-linked with a polyaspartamide derivative (PHEA-EDA) have been used as a substitute for amniotic membrane for delivery of human LECs [16]. Additionally, a 3D HA hydrogel with and without collagen type I has been shown to enhance the growth and differentiation of human corneal epithelial cells (hCECs) when co-cultured with adipose stem cells (hASCs) [17]. Chen et al. [18] have recently developed a HA hydrogel scaffold for cultivation of LSCs in a xenogeneic-free culture system. These authors used a commercially available HyStem^®^-C hydrogel kit, which includes three main components: thiol-modified HA (Glycosil^®^), thiol-reactive polyethylene glycol diacrylate crosslinker (PEGDA; Extralink^®^) and thiol-modified collagen (Gelin-S^®^). A blank-slate matrix form (non-sulfated, without Gelin-S^®^), known as HyStem^®^ Hydrogel kit is also available for applications requiring specific attachment factor optimization. 

HA, also referred to as hyaluronic acid or hyaluronate, is a major component of the extracellular matrix. Non-sulfated HA glycosaminoglycan plays a key role in cell proliferation, growth, survival, polarization and differentiation. The diverse biological roles of HA are linked to the combination of HA-physicochemical properties and HA-binding proteins [19,20,21]. The HA hydrogel is a 3D-network of polymer–polymer and hydrophilic polymer–water molecular interactions. Physical properties of the hydrogel such as viscosity, elasticity, stiffness, shape and structure can be altered by chemical modification [22,23,24]. 

Depending on the type of modification, the resulting derivatives differ significantly in their properties. For example, enzymatic degradation of HA may be affected after chemical alteration, thereby changing non-inflammatory high molecular weight HA to pro-inflammatory low molecular weight HA [25,26]. HA solubility and poor physical properties (e.g., mechanical strength, gel formation time and degradation in vivo) encourage the use of crosslinkers. However, some crosslinkers have been shown to be cytotoxic to stem cells [27,28,29]. Therefore, parameters such as the source and concentration of HA, nature of crosslinker, ratio of HA to crosslinker and buffer environment should be considered when designing HA hydrogel scaffolds for particular cell types. Among these factors, the purity of HA and biosafety of crosslinker are considered critical in clinical applications [24,28,30]. 

The present study aimed to optimize a HA hydrogel scaffold for culture of an OMEC sheet for potential application in LSC therapy. Scaffold preparation began with mixing various ratios of components in the HyStem^®^ hydrogel kit and extending gelation/drying time. Collagen IV was selected for coating the HA hydrogel as it promotes the attachment of stem cells. The optimal mixture was selected by observing cell attachment and initial growth. The influence of the HA hydrogel on OMEC morphology, metabolic activity and expression of 15 genes was investigated following three weeks’ culture. 

## 2. Materials and Methods

### 2.1. Chemicals

Dulbecco’s Modified Eagle Medium/Nutrient F-12 Ham + GlutaMAXTM-I (DMEM/F12) was purchased from Invitrogen Life Technologies (Carlsbad, CA, USA), and the defined proprietary culture medium, CNT-Prime, from Cellntec Advanced Cell Systems AG (Bern, Switzerland). Membrane insert (Transwell cat. no. 3450) and 48-well non-tissue culture polystyrene plate (Falcon 353078) were obtained from Corning Costar (Cambridge, MA, USA) and Becton Dickinson Labware (Franklin Lakes, NJ, USA), respectively. The HyStem^®^ Cell Culture Scaffold kit and ATPlite Luminescence Assay kit were supplied by ESI Bio (Alameda, CA, USA) and Perkin Elmer (Boston, MA, USA), respectively. All other chemicals were purchased from Sigma Aldrich (Oslo, Norway). All reagents were of the highest commercial grade available.

### 2.2. Ethical Considerations

Prior to OMEC isolation from cadaveric donors, local ethical approval and informed consent were obtained from relatives. All procedures performed in this study were in compliance with the Declaration of Helsinki. The use of experimental protocols for the isolation and use of OMECs has been reviewed by The Regional Committee for Medical and Health Research Ethics, Section C, South East Norway (reference: 2017/418). 

### 2.3. Cell Isolation and Culture Conditions

The biopsies were collected from lower labial of cadavers’ oral cavity within 24 h postmortem, and rinsed with Dulbecco’s Modified Eagle Medium/Nutrient F-12 Ham + GlutaMAXTM-I (DMEM/F12) supplemented with 100 U/mL P/S. The biopsies were cut into pieces (1 cm × 0.5 cm) and incubated in 1.2 U/mL dispase II in Mg^2+^ and Ca^2+^-free Hanks’ balanced salt solution at 37 °C overnight. The epithelial cell layer was separated from the lamina propria layer under a dissecting microscope using sterile forceps and scalpel followed by rinsing with DMEM/F12. The tissue was cut into 1–3 mm^2^ explants, placed on plastic inserts and allowed to attach in culture medium (DMEM/F12, 10% fetal bovine serum and penicillin-streptomycin (PenStrep)). Subsequently, the cells were cultured in a humidified incubator at 37 °C, containing 5% CO_2_ for 2 weeks. After 2 weeks, the expanded cells were harvested for experiments as passage 1 (P1) cells using 0.25% trypsin-EDTA solution. 

The viability and number of P1 cells were evaluated by the trypan blue exclusion test. The OMECs were seeded on HA hydrogel scaffolds in serum-free CNT-Prime medium supplemented with PenStrep on a 48-well-plate. All the cultures were incubated at 37 °C and 5% CO_2_. The culture medium was changed every 2–3 days. Cells seeded on collagen IV-coated plastic multiwell plates were included as a control in all experiments. 

### 2.4. Preparation of HA Hydrogels

According to the manufacturer’s instructions, degassed deionized (DG) water was injected into the supplier’s flask containing lyophilized glycosil powder using a sterile syringe under a sterile laminar-flow hood. The mixture was left on a roller for 30–45 min at room temperature to completely dissolve the powder. For Extralink, the reconstitution was instant once DG water was added. The ratio of Glycosil and Extralink used to make mixtures 1–5 are listed in Table 1. 

The 48-well non-tissue culture-treated polystyrene plates were used to promote adherence of the gel to the well and to promote optimal gel formation [31]. Two hundred µL (~2 cm height) of gel mixture was added to each well. The plate was left under a sterile laminar-flow hood to dry. Approximately 200 µL of 1 mg/mL or 20 µg/mL collagen IV was used to coat the HA hydrogel scaffolds after required gelation/drying time and left overnight at 4 °C. For ≥ 3 days gelation/drying time, plates were kept under a sterile laminar-flow hood at room temperature for the first 48 h and then transferred to 4 °C for the remainder of the drying period. Scaffolds were carefully washed with PBS prior to cell seeding. 

### 2.5. Experimental Design

The summary of experiments is shown in Table 2 and Figure S1. In the first set of experiments, HA hydrogel scaffolds were prepared by mixing various ratios of components in the HyStem^®^ hydrogel kit (Table 1) to study any toxic effects of the crosslinker (PEGDA) at different concentrations. Cell attachment and morphology of OMECs were observed after 1 day of culture on uncoated and coated scaffolds (collagen IV 1 mg/mL and 20 µg/mL). 

In the second set of experiments, the gelation/drying time of mixture 5 was extended to 1 and 2 days before coating with 1 mg/mL and 20 µg/mL collagen IV. The scaffolds were used to study cell attachment and morphology of OMECs after 1 and 3 days of cell culture. 

In the third set of experiments, the gelation/drying time of mixture 5 was extended to 3, 7, 10 and 14 days prior to being coated with 1 mg/mL collagen IV to study cell attachment and morphology of OMECs after 1, 3, 7, 10 and 14 days of cell culture. Three technical replicates were used in each set of above-mentioned experiments.

Finally, mixture 5 with 3-day gelation/drying time was coated with 1 mg/mL collagen IV and used to study cell attachment and morphology after 1, 3, 7, 10, 14 and 21 days of cell culture. Cell metabolic activity and expression of 15 genes signifying stem cell phenotype were assessed after three weeks. 

### 2.6. Cell Morphology 

Cell morphology and the integrity of the complete cell layer were examined using a Leica DMIL inverted phase contrast microscope (Leica Microsystems, Wetzlar, Germany) equipped with a Canon EOS 5D mark II camera (Canon, Oslo, Norway). The images were captured at random positions within each well. 

### 2.7. Cell Metaboilc Activity 

Measurements were performed using an ATPlite kit following the manufacturer’s instructions. In short, cell lysis buffer was added to cultures and left on a shaker for 5 min in the dark. The luminescent substrate was then added, covered in foil and left on a shaker for 10 min in the dark. Thereafter, the contents were transferred from 48-well culture plates to white plates. Luminescence was read in a Victor3™ 1420 Multilabel Counter (Perkin-Elmer Life Sciences, Wiesbaden, Germany). Three technical replicates were used. The blank as a measure of background was subtracted from experimental values. 

### 2.8. RNA Extraction, cDNA Synthesis, Real-Time Quantitative Polymerase Chain Reaction (RT-qPCR) and Data Analysis

The cells were harvested from each well by adding RLT buffer (Qiagen, Hilden, Germany) and mixed by pipetting. Thereafter, the lysate was passed through a 0.9-mm diameter needle attached to a sterile plastic syringe 10 times to achieve a homogenous lysate. Total RNA was then extracted and purified using RNeasy micro kit (Qiagen, Hilden, Germany), according to the manufacturer’s instructions. Purity and quantity of isolated RNA were measured by spectrophotometry (Nanodrop, Wilmington, Germany). Reverse transcription (RT) was performed using the High-Capacity cDNA RT Kit (Applied Biosystems, Abingdon, UK) with 200 ng of total RNA per 20 µL RT reaction. Comparative relative quantification was performed using the StepOnePlus^TM^ Real-Time polymerase chain reaction (PCR) system (Applied Biosystems) and Taqman Gene Expression assays following protocols from the manufacturer (Applied Biosystems) for 15 genes (*ABCG2*, *ΔNp63α*, *ALDH1L2*, *CDH1*, *CDH2*, *GJA1*, *Vimentin*, *ITGAV*, *ITGA6*, *ITGB4*, *ITGB5*, *CASP3*, *BAX2*, *BCL2* and *PCNA*) (Table 3). All the samples were run in triplicates (each reaction: 2.0 µL cDNA, total volume 20 µL). The thermo cycling parameters were 95 °C for 10 min followed by 40 cycles of 95 °C for 15 s and 60 °C for 1 min. 

The average *E* of the polymerase chain reaction (PCR) was calculated for each target gene from triplicates of the two extreme samples, based on cycle threshold values (Ct) [32]. The acceptable range of efficiencies was between 0.7 and 1.0. Expression levels relative to a 1× (control, calibrator) sample were then calculated by the ΔΔCt method adjusted for *E*. Gene expression data were analyzed using the Relative Expression Software Tool (REST^©^, Relative Expression Software Tool, Weihenstephan, Germany) that implements the Pair Wise Fixed Reallocation Randomization Test^©^ to investigate the significance of changes in gene expression [33]. The gene expression data were presented as fold change compared to untreated sample. Differences were considered significant when *p* < 0.05, if not stated otherwise. Data are presented as the mean ± standard error. 

### 2.9. Statistical Analysis

The t-test was used in the analysis of results from the ATPlite assay [34,35]. A *p* value of <0.05 was considered to be significant. Statistical analysis and graph preparation were performed using GraphPad Prism 6.0 for Mac (GraphPad Software, San Diego, CA, USA). Cell metabolic activity data are presented as the mean ± standard error. 

## 3. Results 

### 3.1. Observation of Cells on Initial HA Hydrogel Mixtures Indicate Cytotoxic Effect of Crosslinker

**Figure 1:**
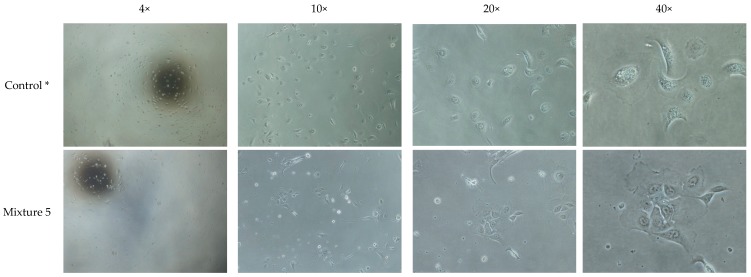
Light microscope images (4×, 10×, 20× and 40× magnification) of 1-day old culture of human OMECs on HA hydrogel scaffold mixture 5 coated with 1 mg/mL collagen IV. Cell attachment and morphology of OMECs on mixture 5 were better than other hydrogel scaffold mixtures. Therefore, it was selected for further optimization. * Cultured human OMECs on 1 mg/mL collagen IV coated plastic 24-well-plate.

Our initial investigation of five different HA hydrogel mixtures that were left uncoated or coated with collagen IV at two different concentrations (20 µg/mL or 1mg/mL) showed OMEC attachment and morphology at one day of culture was best on HA hydrogel mixture 5 coated with 1 mg/mL collagen IV (Figure S2; Table 1). OMECs cultured on HA hydrogel mixtures that included the PEGDA crosslinker supplied with the HyStem^®^ hydrogel kit performed poorly (Figure S2; Table 1). Likewise, mixture 5 coated with a lower concentration of collagen IV (20 µg/mL) did not perform well. Cells in these groups had mainly rounded morphology. Cells detached after one day and were washed away during medium change the next day (Figure S2). Thus, HA hydrogel mixture 5 coated with 1 mg/mL collagen IV was selected (Figure 1) for further optimization. 

### 3.2. Extended Drying Time Improves the Integrity of HA Hydrogel Mixture 5

**Figure 2:**
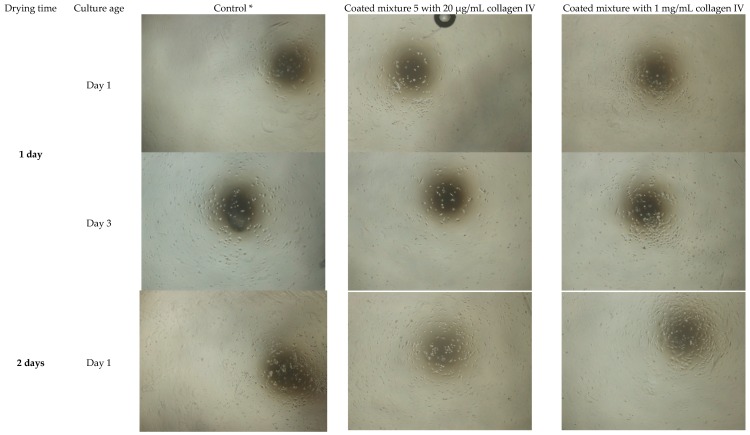

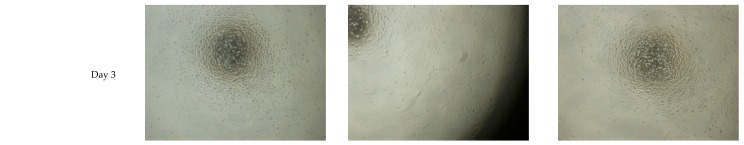
Light microscope images (4× magnification) of 1 and 3 days old cultured human OMECs on HA hydrogel scaffold mixture 5 dried for 1 or 2 days and coated with 1 mg/mL and 20 µg/mL collagen IV. OMECs showed improved attachment and morphology when the gelation/drying time of mixture 5 HA hydrogel was increased to two days. * Cultured human OMECs on coated plastic 24-well-plate with 1 mg/mL collagen IV.

**Figure 3:**
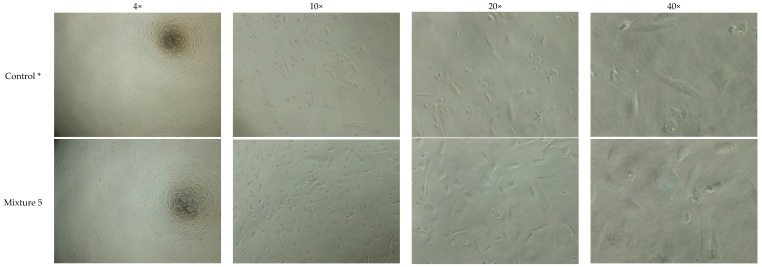
Light microscope images (4×, 10×, 20× and 40× magnification) of human OMECs after 3 days of culture on HA hydrogel scaffold mixture 5 dried for 3 days and coated with 1 mg/mL collagen IV. It was the best attachment and cell density compared to other tested gelation/drying times of mixture 5 (i.e., 3, 7, 10 and 14 drying days). * Cultured human OMECs on coated plastic 24-well-plate with 1 mg/mL collagen IV.

OMECs showed improved attachment and morphology when the gelation/drying time of mixture 5 HA hydrogel was increased to two days. HA hydrogels coated with 1 mg/mL collagen IV performed best (Figure 2). However, we saw patches of detached floating HA hydrogel scaffold containing cells in the culture medium, indicating the structural integrity of the HA hydrogel could be improved (data not shown). Therefore, we tested various gelation/drying times of mixture 5 up to two weeks (i.e., 3, 7, 10 and 14 drying days) to improve the integrity of the hydrogel while maintaining cell attachment and growth. Observation of OMEC attachment and morphology during this culture period indicated best attachment and cell density using HA hydrogel mixture 5 dried for three days and coated with 1 mg/mL collagen IV (3-HAC4) (Figure 3; Figure S3). 

Based on these results 3-HAC4 was selected for further analysis. Cell morphology, metabolic activity and expression of 15 genes of OMECS were investigated following a 3-week culture period, which is the typical culture time required to produce cell sheets suitable for transplantation to the eye.

### 3.3. HA Hydrogel Supports Cell Adhesion of Three-Week Culture of Oral Mucosal Cells

**Figure 4:**
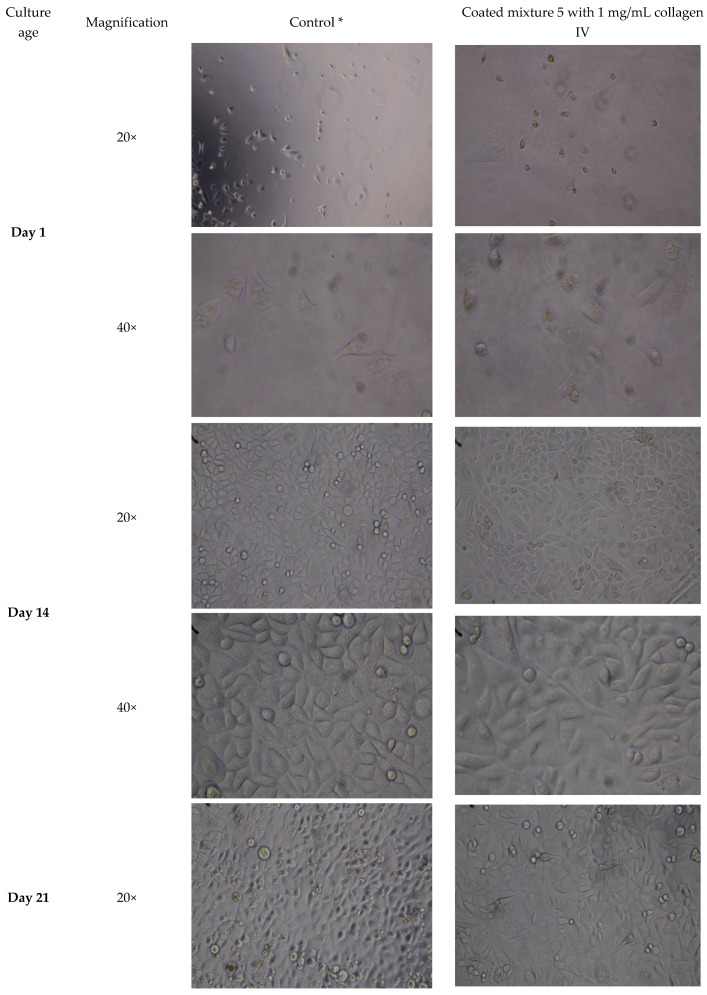

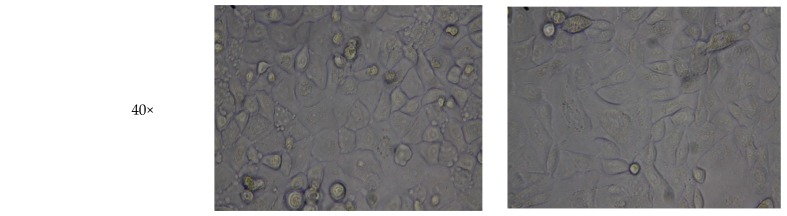
Light microscope images (20× and 40× magnification) of human OMECs after 1, 14 and 21 days of culture on 3-day old HA hydrogel scaffold mixture 5 coated with 1 mg/mL collagen IV. A 3-week culture of OMECs resulted in a full sheet of cells with morphological features resembling control cells. * Cultured human OMECs on coated plastic 24-well-plate with 1 mg/mL collagen IV.

**Figure 5:**
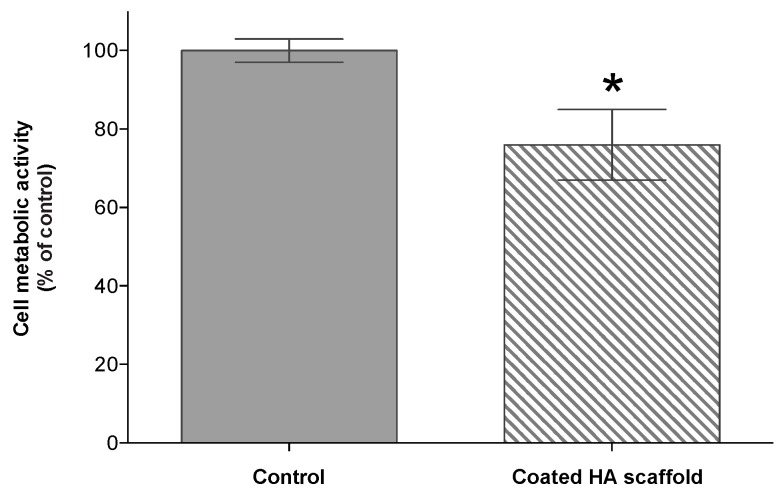
Metabolic activity of human OMECs after three weeks of culture on 3-day old HA scaffold coated with 1 mg/mL collagen IV. Asterisk (*) above the bar denotes significant differences (*p* ≤ 0.05) as compared with the control (seeded cells on coated multiwall plates with collagen IV).

A 3-week culture of OMECs on 3-HAC4 resulted in a full sheet of cells with morphological features resembling control cells cultured on collagen IV coated plastic (Figure 4 and Figure S4). The ATPlite luminescence assay indicated significantly lower metabolic activity in the HA hydrogel scaffold group compared to control after three weeks of cell culture (76%; *p* ≤ 0.05; Figure 5). 

### 3.4. HA Hydrogel Promoted Increased Expression of Stem Cell Markers and Cell Adhesion Genes in Three-Week Cultures of Oral Mucosal Cells

**Figure 6:**
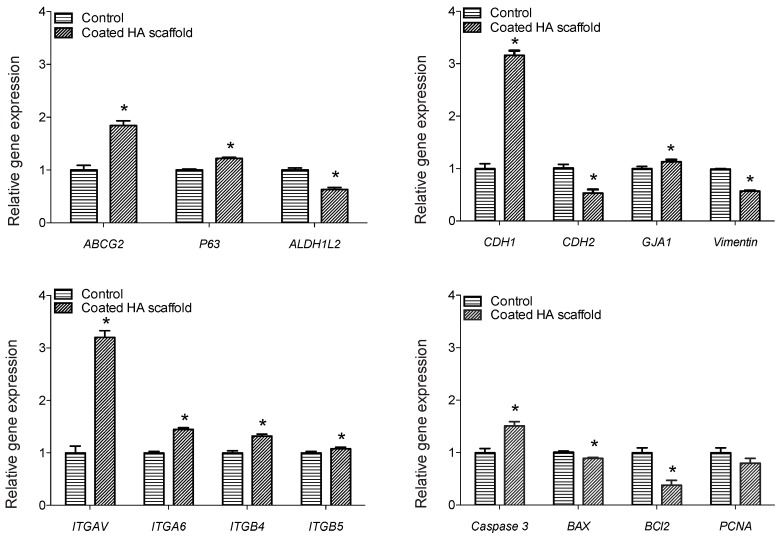
Real-time polymerase chain reaction (RT-PCR) analysis of the expression of 15 genes (*ABCG2*, *ΔNp63α*, *ALDH1L2*, *CDH1*, *CDH2*, *GJA1*, *Vimentin*, *ITGAV*, *ITGA6*, *ITGB4*, *ITGB5*, *CASP3*, *BAX2*, *BCL2* and *PCNA*) in human OMECs after three weeks of culture on 3-day old HA scaffold coated with 1 mg/mL collagen IV relative to control (seeded cells on collagen IV coated plastic multiwall plates), which was chosen as calibrator. Asterisks (*) above the bar denote significant differences (*p* ≤ 0.05).

The expression of 15 genes was investigated in OMEC sheets cultured on 3-HAC4 for three weeks compared to control cells cultured on collagen IV coated plastic. Significant upregulation was observed for *ABCG2* (1.84-fold), *ΔNp63α* (1.22-fold), *CDH1* (3.16-fold), *GJA1* (1.13-fold), *ITGAV* (3.20-fold), *ITGA6* (1.45-fold), *ITGB4* (1.32-fold), *ITGB5* (1.08-fold) and *CASP3* (1.51-fold) compared to control (*p* ≤ 0.05; Figure 6 and Table 4). Five genes, namely *ALDH1L2* (1.59-fold), *CDH2* (1.90-fold), *Vimentin* (1.74-fold), *BAX2* (1.13-fold) and *BCL2* (2.63-fold), were significantly downregulated between two groups at the RNA level (*p* ≤ 0.05; Figure 6 and Table 4). Although *PCNA* was downregulated but there was no significant difference compared to control (*p* ≥ 0.05; Figure 6). 

## 4. Discussion 

Our results indicate that 3-HAC4 can be used to culture a full sheet of OMECs. The morphological features of the cells were comparable to that of the control following three weeks of culture. Additionally, the expression of genes associated with stem cell (*ABCG2* and *ΔNp63α*), gap junction (*GJA1*) and integrins (especially *ITGAV*) may improve transplantation success. HA hydrogel scaffolds have previously been shown to support culture of hCECs, [36,37], human LECs [16] and co-culture of hCECs with human adipose stem cells when constructed as a 3D HA hydrogel [17]. Our work adds to these studies showing that HA hydrogel scaffolds have potential as a carrier for future applications in ocular surface reconstruction using an alternative cell type. 

The current study found that PEGDA crosslinker, one of the components in the HA hydrogel mixture supplied in the HyStem^®^ hydrogel kit, had a cytotoxic effect on cultured OMECs. No such effect was observed by Chen et al. [18] when they used PEGDA during development of a HA hydrogel scaffold for ex vivo culture of limbal stem cells in a xenogeneic-free culture system. Crosslinkers are commonly used to overcome the poor solubility and physical properties of HA hydrogels, such as mechanical strength and gel formation time, but they may have a cytotoxic effect on some types of stem cells [27,28,29]. The biosafety and purity of HA hydrogels are critical factors in the design of materials for clinical application [28,30]. In the absence of PEGDA, the gel formation of dissolved Glycosil^®^ powder in water would eventually occur due to auto-crosslinking of the thiol-modified HA. However, the gelation time is longer and extended drying time is needed to increase rigidity.

OMECs showed better attachment and morphology when the HA hydrogel scaffold was coated with 1 mg/mL collagen IV. Collagen is the most abundant and key structural fibrous protein in the extracellular matrix (ECM). It regulates cell adhesion, supports chemotaxis and migration, and directs tissue development [38]. Among the 29 collagen proteins identified, collagen IV is the major scaffolding component of basement membranes, where its self-assembled ropelike networks are formed to support adhesion of stem cells [39,40]. The adhesion mechanism is proposed to be through α1β1, α2β1, α10β1 and α11β1 integrins that are responsible for linking the ECM with the intracellular cytoskeleton [41,42]. For example, high expression level of *ITGB1* was shown to be correlated with high proliferation capacity in human oral keratinocytes [43]. It was proposed that its higher affinity to collagen IV results in enrichment of cells residing in the basal layer adhering to the basement membrane such as stem cells. In addition to OMECS [44], similar methods have been used in the past to enrich human epidermal stem cells [45,46,47]. The coating of substrates for cell culture is commonly performed by incubating a solution of collagen IV on the material for several hours to deposit a layer, followed by washing off the excess solution. Factors such as hydrophilicity of substrate and surface charge play important roles in formation of collagen IV layers by solution deposition method [39,48,49]. 

According to our ATPlite luminescence assay the metabolic activity of cells was reduced to 76% of the control. This may reflect the gene expression results suggesting some cells had entered apoptosis. Alternatively, ~3-fold upregulation of *CDH1* shown in our RT-qPCR results may account for the decreased metabolic activity of OMECs on the HA hydrogel. As well as cell-cell adhesion, *CDH1* acts as a tumor suppressor protein, preventing cells from growing and dividing too rapidly or in an uncontrolled way [50]. Of apoptosis-related genes, the pro-apoptotic markers *CASP3* and *BAX2* were upregulated and downregulated, respectively, compared to control whereas the anti-apoptotic marker *BCL2* was downregulated in OMECs cultured on 3-HAC4. This suggests that some cells may have entered the apoptosis pathway. However, *PCNA* expression was unchanged, suggesting that cell proliferation was similar to control. 

Importantly, after three weeks OMEC culture on 3-HAC4, significant upregulation was seen in the expression levels of stem cell associated genes *ABCG2* and *ΔNp63α* compared to control. They have previously been shown as stem cell markers for OMECs [51,52]. However, *ALDH1L2* had significantly lower expression. There is growing evidence to suggest *ALDH* superfamily not only being used as stem cell marker but also regulate cellular functions associated with self-renewal, expansion and differentiation [53]. Thus, our selected HA hydrogel formula promoted upregulation of stem cell-associated markers compared to OMECs cultured using a standard culture method. Transplantation of cultured LSC sheets containing a high number of cells with *ΔNp63α* expression signifying the stem-cell phenotype has been correlated to clinical success in treatment of LSCD [14]. The authors reported successful transplantation in 78% of patients when LSCs cultured on fibrin expressed more than 3% *ΔNp63α*. 

Among genes for adhesion-associated (cadherins and integrins), gap junction (*GJA1*) and intermediate filament (*Vimentin*) proteins, significant upregulation was observed for *CDH1*, *GJA1*, *ITGAV*, *ITGA6*, *ITGB4* and *ITGB5* whereas downregulation for *CDH2* and *Vimentin* compared to the control. Cadherins and integrins are involved in cell–cell and cell–matrix interactions. They mediate a number of crucial processes in stem cells including cell survival, proliferation, self-renewal and differentiation [54]. Increased expression of *CDH1* has been shown in rabbit OMEC sheet grafts compared to normal and LSCD epithelium [55]. Authors suggested that these results implied strong adhesion between cells, which may protect the ocular surface following transplantation. Notably, expression of *ITGAV* has been shown in oral mucosal wounds where it is associated with scar-less wound healing [56]. Thus, the ~three-fold increase in expression of *CDH1* and *ITGAV* along with gap junction (*GJA1*) and other integrins (*ITGA6*, *ITGB4*, and *ITGB5*) in our OMEC 3-HAC4 cultures could be beneficial for protection and scar-less healing of the ocular surface following transplantation. 

The present study was designed to optimize a novel HA hydrogel scaffold for culture of OMECs for potential application in LSCD therapy. The results indicate that coating HA with collagen IV and including a 3-day drying time in the absence of PEGDA crosslinker, leads to a hydrogel that supports growth of a full sheet of OMECs after three weeks of culture. Further research might investigate the application of alternative crosslinkers, e.g., polyvinyl alcohol. Use of an OMEC biocompatible crosslinker would overcome the floppy appearance, improve the integrity and stability of the hydrogel and eliminate the need for a 3-day drying period. The morphological features of OMECs were comparable to the control. In addition, 3-HAC4 promoted expression of genes that may improve transplantation success, *ABCG2*, *ΔNp63α CDH1*, *GJA1* and integrins (especially *ITGAV*). 

## Figures and Tables

**Table 1:** The ratios of components in preparing hyaluronan (HA) hydrogel scaffolds for the first set of experiments.

	Mixture 1	Mixture 2	Mixture 3	Mixture 4	Mixture 5
Glycosil	80%	60%	40%	20%	100%
Extralink	20%	40%	60%	80%	0
Gelation time *	30 min	30 min	30 min	~6h	~24h
Appearance	Firm	Firm	Firm	Little floppy	Floppy

Glycosil: Thiol-modified HA and Extralink: Thiol-reactive polyethylene glycol diacrylate (PEGDA) crosslinker. * At room temperature.

**Table 2:** Summary of experiments.

Study No.	Objective	Endpoint	Examination/Assay	Hyaluronan (HA) Scaffold			Culture Age
				Formula *	Drying Time	Collagen Coating	
1)	To study toxic effects of crosslinker and compare collagen IV coatings	Cell attachment and morphology	Light microscope	Mixtures 1–5	30 min to ~1 day	Uncoated and coated (1 mg/mL and 20 µg/mL)	1 day
2)	To study effects of 1- and 2-day old collagen coated HA scaffold on oral mucosal epithelial cells (OMECs) culture within 3 day	Cell attachment and morphology	Light microscope	Mixture 5	1 and 2 days	Coated (1 mg/mL and 20 µg/mL)	1 and 3 days
3)	To study effects of 3- to 14-day-old collagen coated HA scaffold on OMECs cultured for 2 weeks	Cell attachment and morphology	Light microscope	Mixture 5	3, 7, 10 and 14 days	Coated (1 mg/mL)	1, 3, 7, 10 and 14 days
4)	To study effects of 3-day old collagen-coated HA scaffold on OMECs cultured for 3 weeks	Morphology, metabolic activity and gene expression	Light microscope, ATPlite luminescence and real-time quantitative polymerase chain reaction (RT-qPCR) ^≠^	Mixture 5	3 days	Coated (1 mg/mL)	1, 3, 7, 10, 14 and 21 days ^≠^

^*^ The various ratios of components for mixtures 1–5 are listed in Table 1. ^≠^ OMEC morphology (light microscope) was observed at 1, 3, 7, 10, 14 and 21 days of culture whereas assessment of metabolic activity (ATPlite luminescence) and expression of selected genes (RT-qPCR) was carried out at 21 days of culture. OMEC control was included in all experimental setups (OMEC on collagen IV coated multiwall plates).

**Table 3:** List of primers used in gene expression analyses.

Gene Symbol	Gene Name	Alias	Taqman Assay ID	*E*-value
*GAPDH*	Glyceraldehyde-3-phosphate dehydrogenase	*G3PD*, *HEL-S-162eP*	Hs99999905_m1	2.00
*ABCG2*	ATP-binding cassette sub-family G member 2	*ABC15*, *ABCP*	Hs01053790_m1	2.07
*ΔNp63α*	Tumor protein p63	*TP63*, *TP53L*	Hs00978343_m1	1.91
*ALDH1L2*	Aldehyde dehydrogenase 1 family member L2	*mtFDH*	Hs00402876_m1	2.00
*CDH1*	Cadherin 1, type 1, E-cadherin	*CD324*, *CDH1*	Hs01023894_m1	2.02
*CDH2*	Cadherin 2, type 1, N-cadherin	*CD325*, *NCAD*	Hs00983056_m1	2.00
*GJA1 (Connexin 43)*	Gap junction protein, alpha 1, 43kDa	*CX43*, *GJAL*	Hs00748445_m1	2.07
*Vimentin*	Vimentin	*Vim, FLJ36605*	Hs00185584_m1	1.97
*ITGAV*	Integrin alpha V	*CD51*, *MSK8*	Hs00233808_m1	2.00
*ITGA6*	Integrin alpha 6	*CD49f*, *VLA-6*	Hs01041011_m1	1.89
*ITGB4*	Integrin beta 4	*CD104*	Hs00236216_m1	1.84
*ITGB5*	Integrin beta 5	-	Hs00174435_m1	2.00
*CASP3*	Caspase 3, apoptosis-related cysteine peptidase	*CPP32*, *CPP32B*	Hs00234387_m1	2.04
*BAX2*	BCL2-associated X protein	*BCL2L4*	Hs00180269_m1	2.00
*BCL2*	BCL2, apoptosis regulator	*Bcl-2*, *BCL2*	Hs99999018_m1	2.05
*PCNA*	Proliferating cell nuclear antigen	*MGC8367*	Hs00696862_m1	1.95

**Table 4:** The gene expression data as fold change in a 3-week culture of OMECs on 3-HAC4 compared to control OMEC sheets grown on collagen IV-coated plastic.

Gene Symbol	Regulation	Fold Change
*ABCG2*	UP	1.84
*P63*	Up	1.22
*ALDH1L2*	Down	1.59
*CDH1*	Up	3.16
*CDH2*	Down	1.90
*GJA1*	Up	1.13
*Vimentin*	Down	1.74
*ITGAV*	Up	3.20
*ITGA6*	Up	1.45
*ITGB4*	Up	1.32
*ITGB5*	Up	1.08
*CASP3*	Up	1.51
*BAX2*	Down	1.13
*BCL2*	Down	2.63

## Supplementary Materials

The following are available online at https://www.mdpi.com/2306-5354/6/4/97/s1: Figure S1: Workflow of experiments; Figure S2: The light microscopic images (4× magnification) of 1-day old culture of human OMECs on five different mixtures of HA scaffold uncoated and coated with 1 mg/mL and 20 µg/mL collagen IV; Figure S3: The light microscopic images (4× magnification) of human OMECs after 1, 3, 7, 10 and 14 days of culture on HA scaffolds dried for 3, 7, 10 or 14 days and coated with 1 mg/mL collagen IV; Figure S4: The light microscopic images (4×, 10×, 20× and 40× magnification) of human OMECs after 1, 3, 7, 10, 14 and 21 days of culture on HA scaffold dried for 3 days and coated with 1 mg/mL collagen IV.
