# Supplementary material for: A Hyaluronan Hydrogel Scaffold for Culture of Human Oral Mucosal Epithelial Cells in Limbal Stem-Cell Therapy
Source: Bioengineering (Basel). 2019 Oct 23;6(4):97. doi: 10.3390/bioengineering6040097 (PMC6955856; doi:10.3390/bioengineering6040097)
Supplement: Supplementary file 1 [file bioengineering-06-00097-s001.pdf]

Supplementary Materials

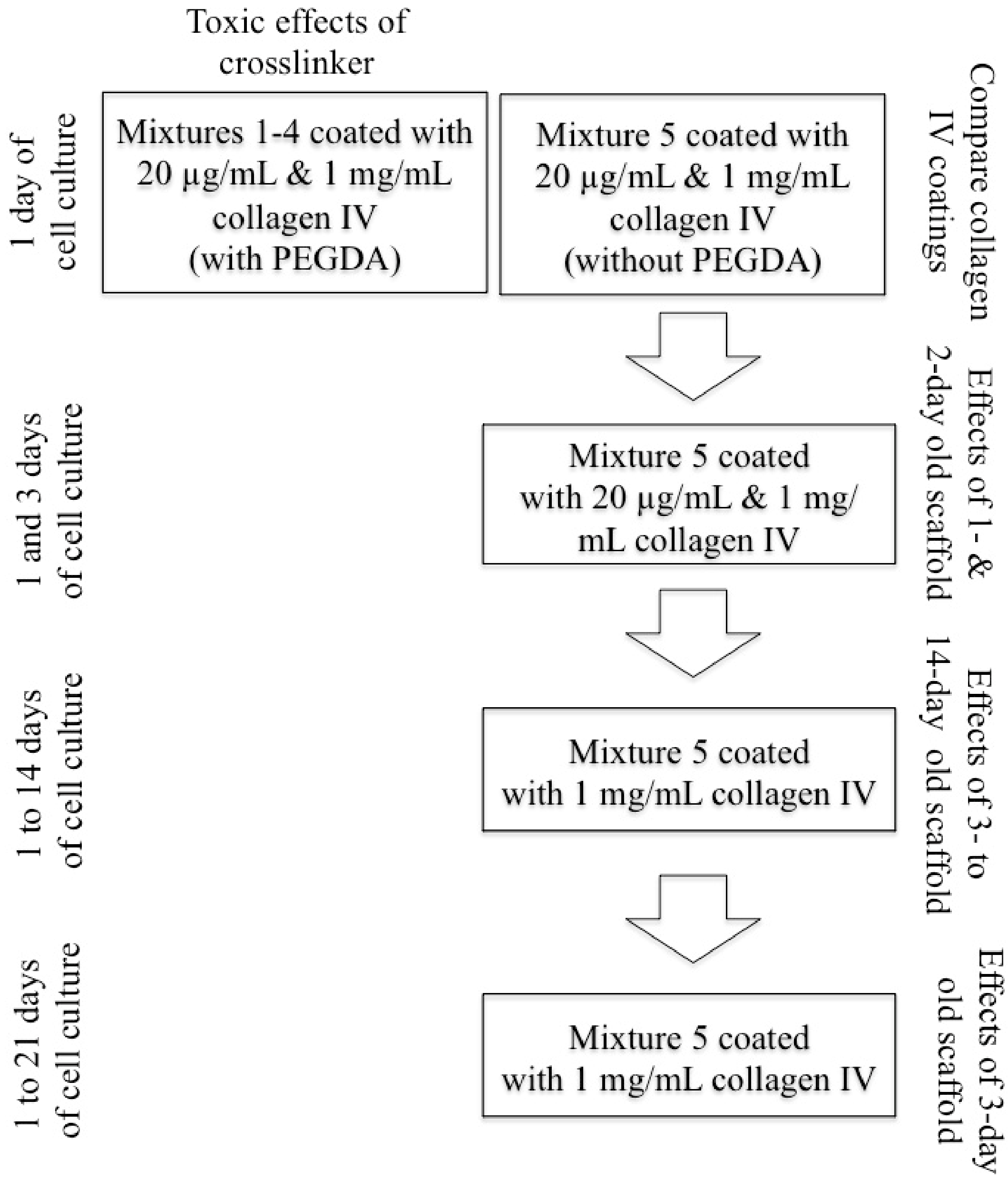

Figure S1. Workflow of experiments.

	Uncoated mixture	Coated mixture with 20 μg/mL collagen IV	Coated mixture with 1 mg/mL collagen IV
Mixture 1	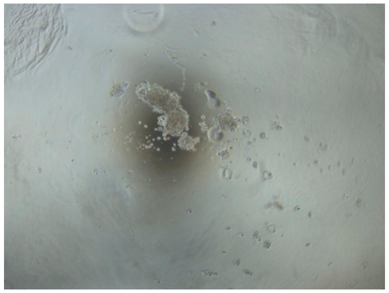	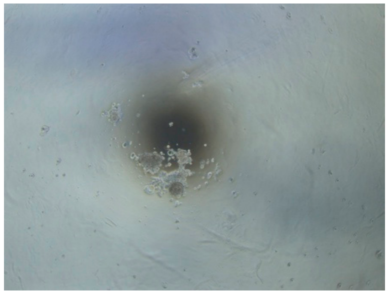	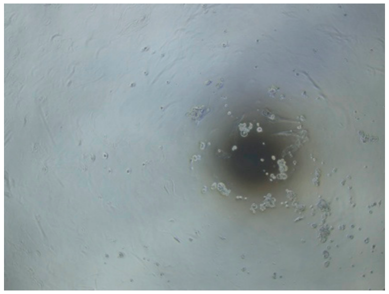
Mixture 2	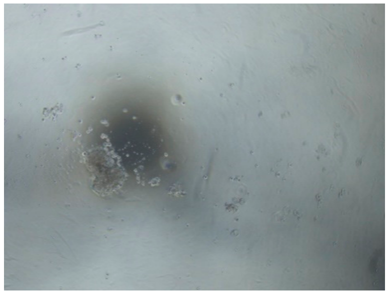	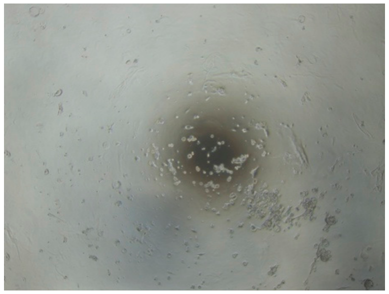	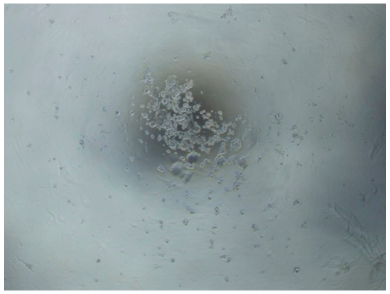
Mixture 3	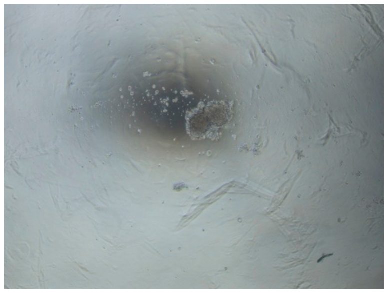	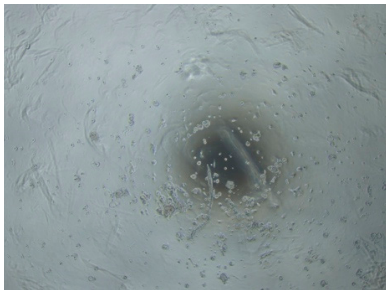	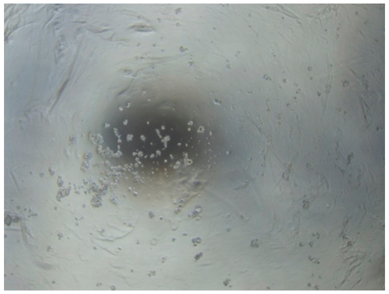

Mixture 4

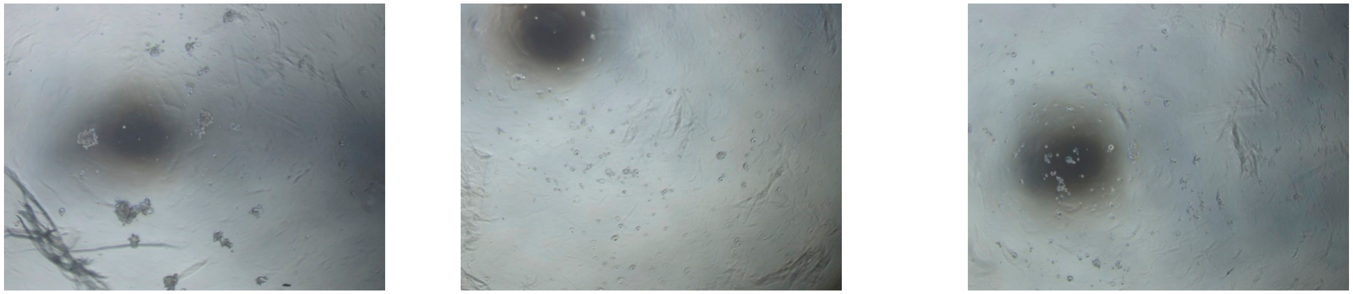

Mixture 5

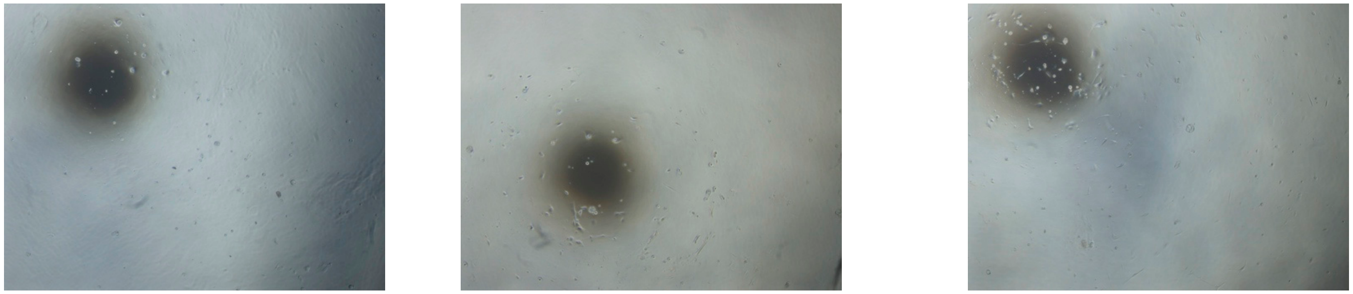

Figure S2. The light microscopic images (4× magnification) of 1-day old culture of human OMECs on five different mixtures of HA scaffold uncoated and coated with 1 mg/mL and 20 μg/mL collagen IV.

Scaffold age	Culture age	Control *	Coated mixture 5 with 1 mg/mL collagen IV
**3 days**			
	Day 1	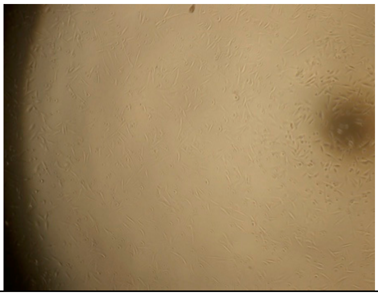	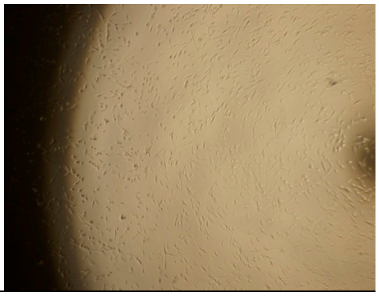
	Day 3	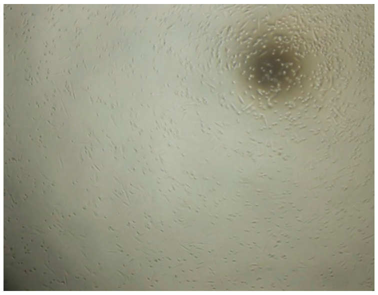	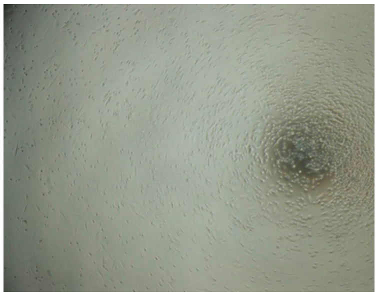
	Day 7	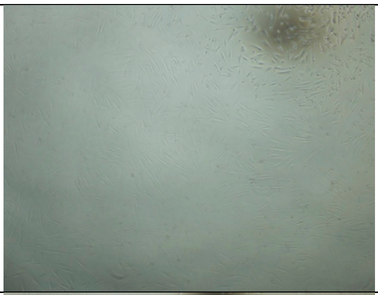	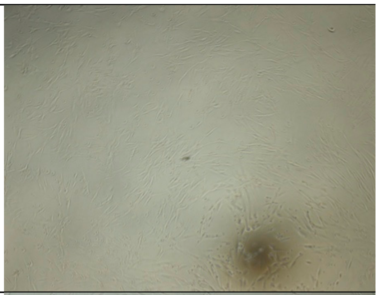
	Day 10	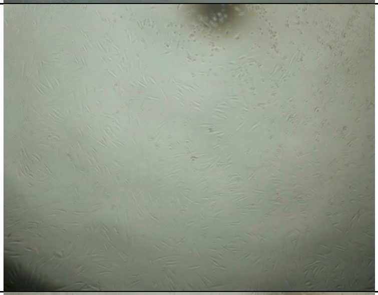	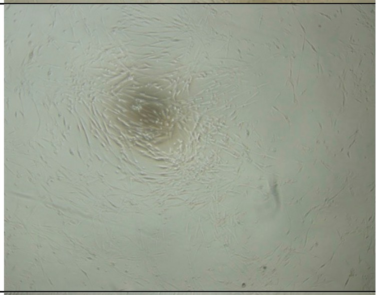
	Day 14	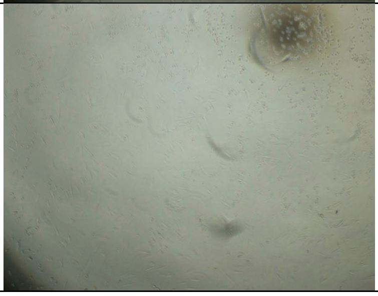	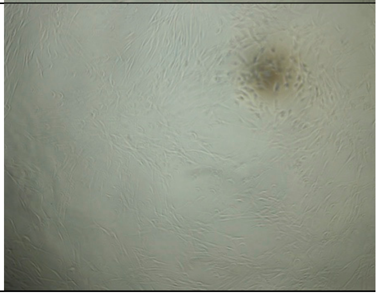
**7 days**			

Day 1	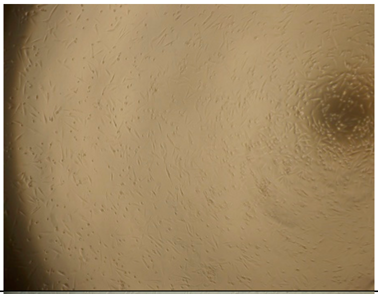	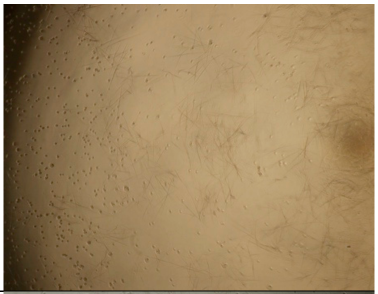
Day 3	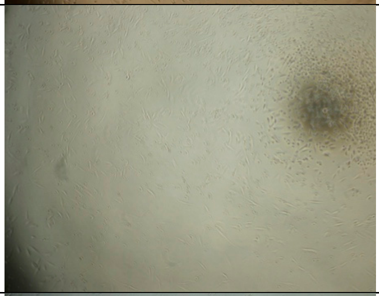	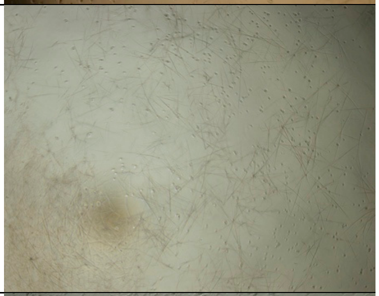
Day 7	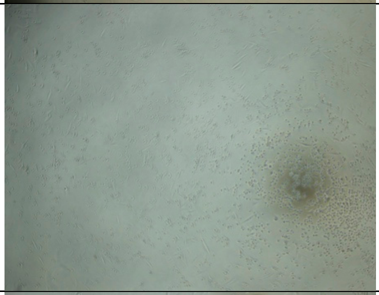	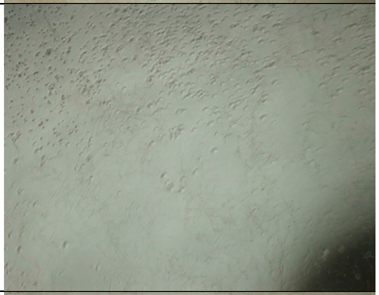
Day 10	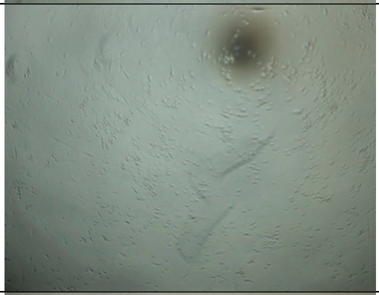	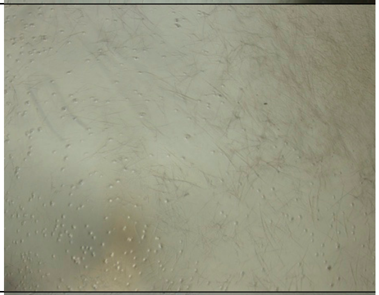
Day 14	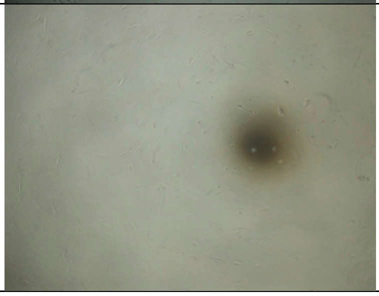	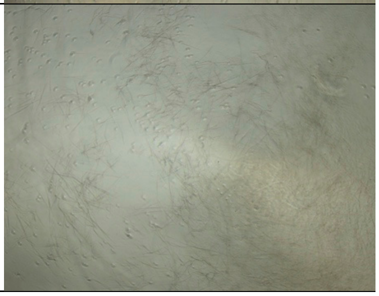

10 days

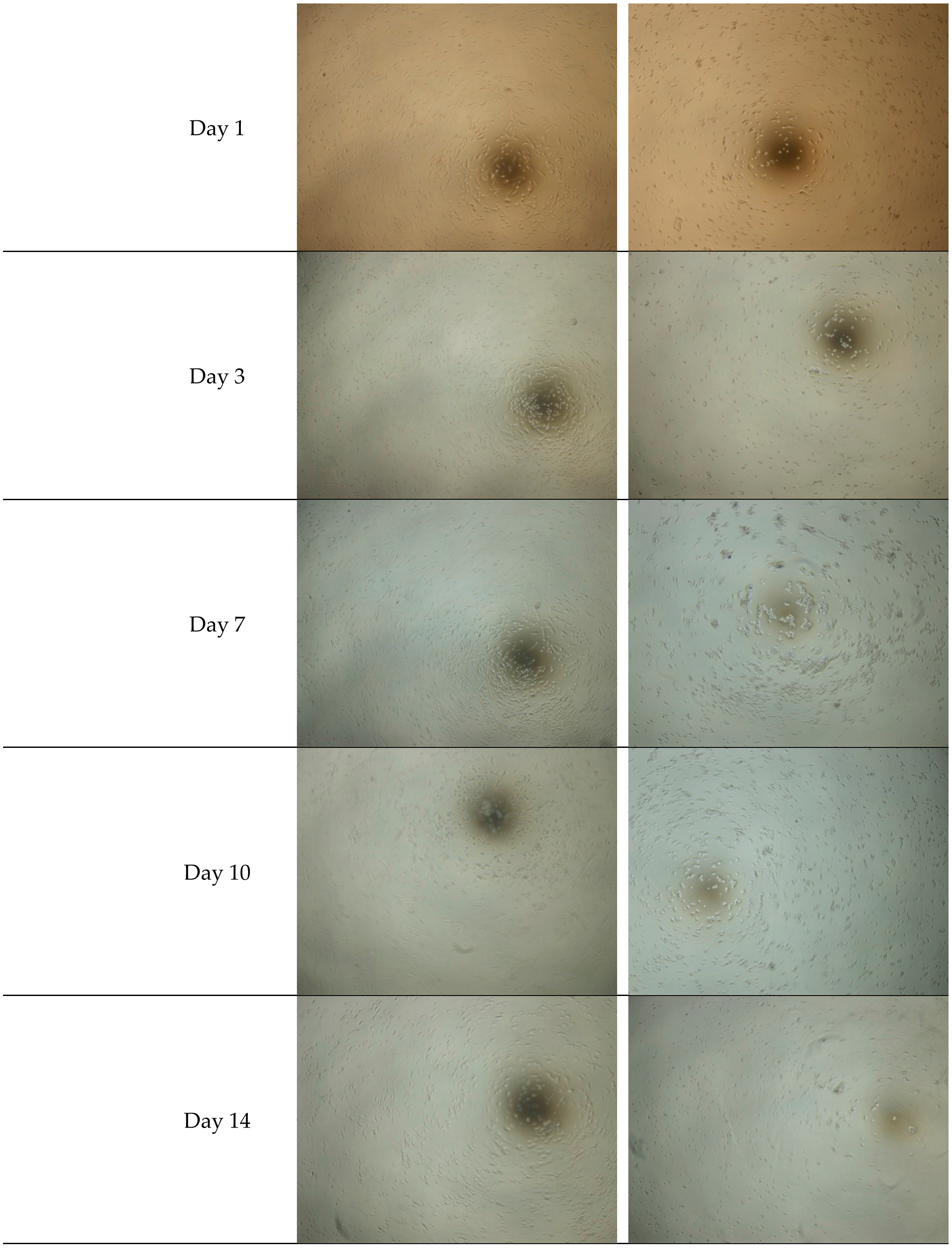

Day 1

Day 3

Day 7

Day 10

Day 14

14 days

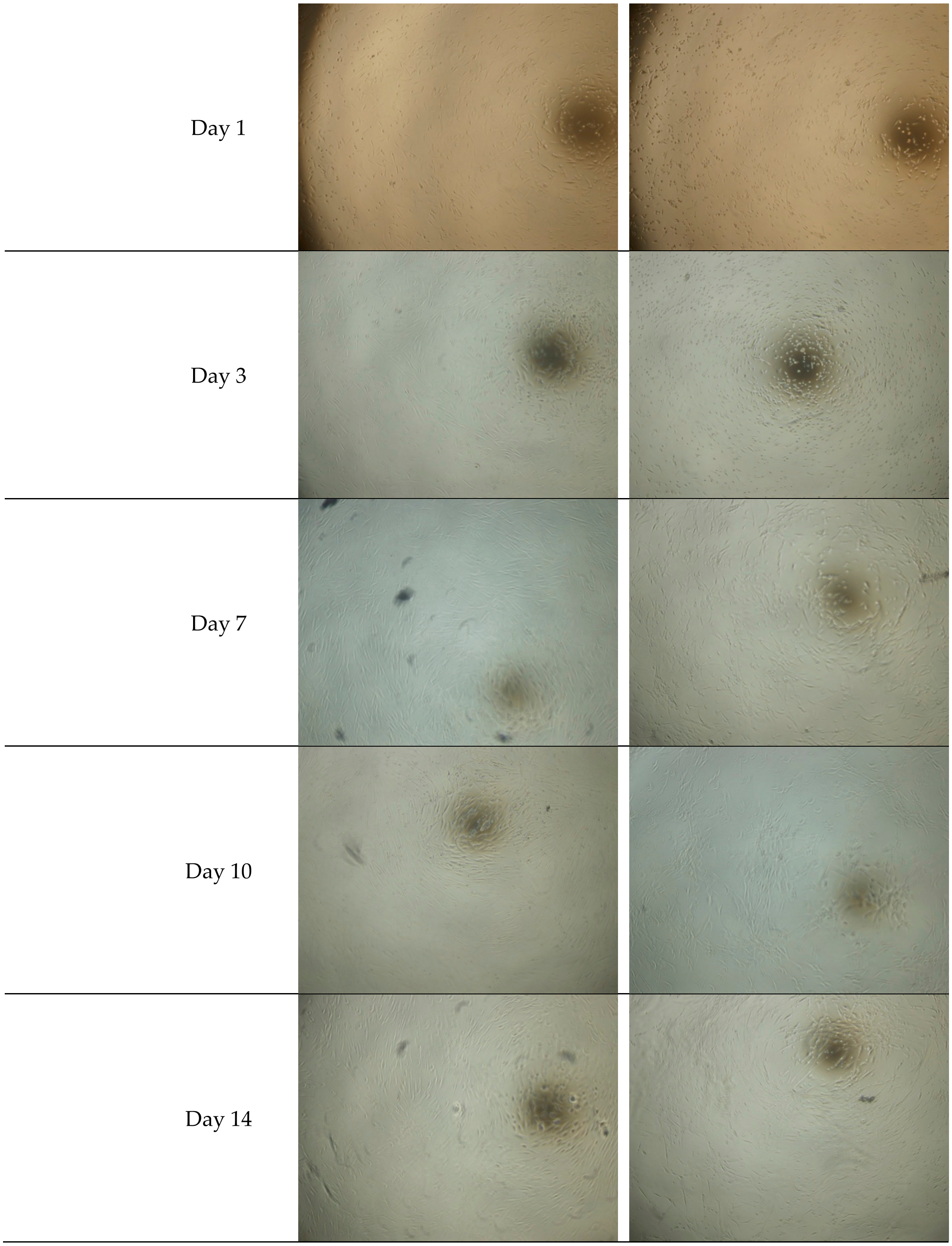

Figure S3. The light microscopic images (4× magnification) of human OMECs after 1, 3, 7, 10 and 14 days of culture on HA scaffolds dried for 3, 7, 10 or 14 days and coated with 1 mg/mL collagen IV. * Cultured human OMECs on coated plastic 24-well-plate with 1 mg/mL collagen IV.

Culture age	Magnification	Control *	Coated mixture 5 with 1 mg/mL collagen IV
**1 day**			
	4×	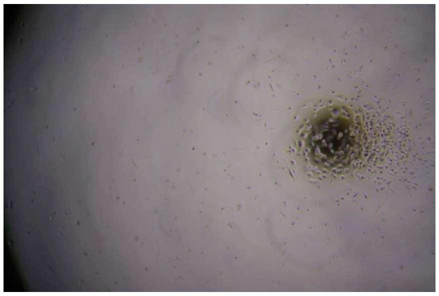	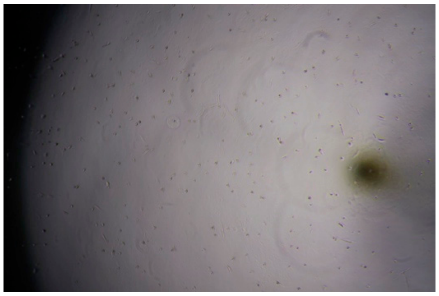
	10×	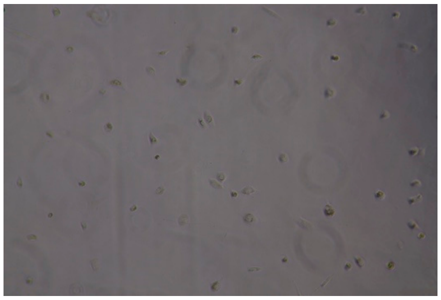	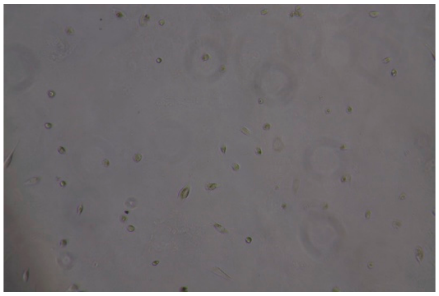
	20×	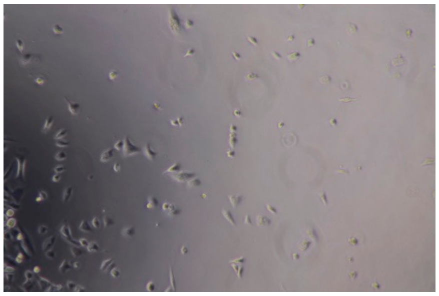	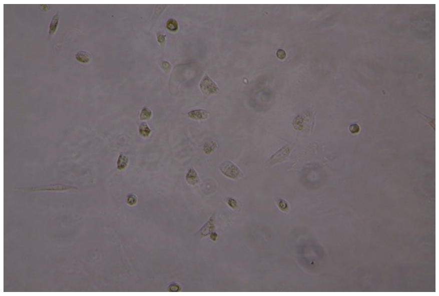
	40×	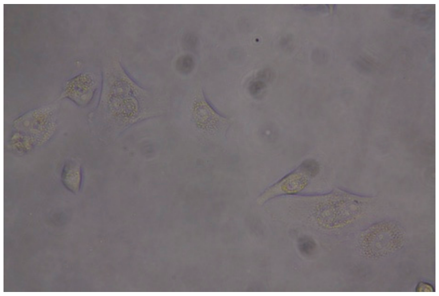	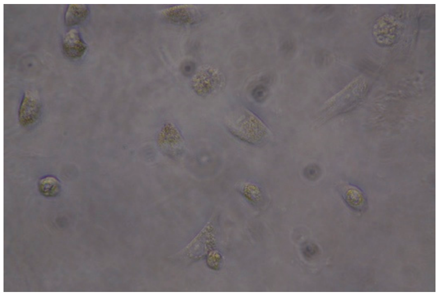
**3 days**			

4×	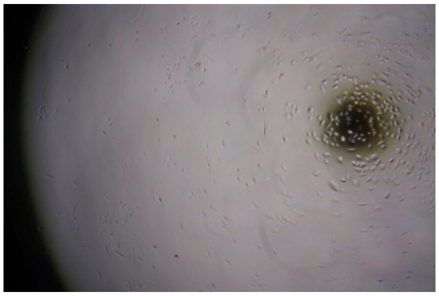	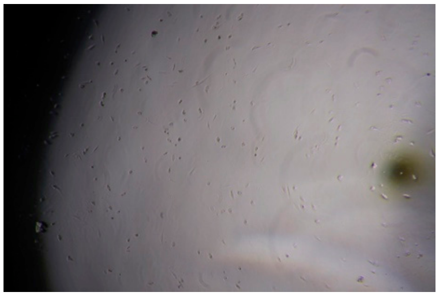
10×	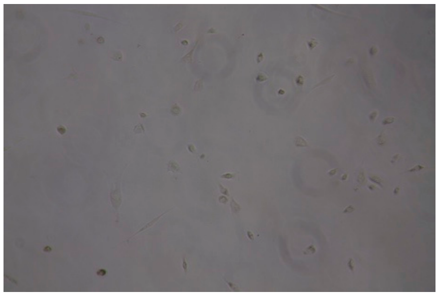	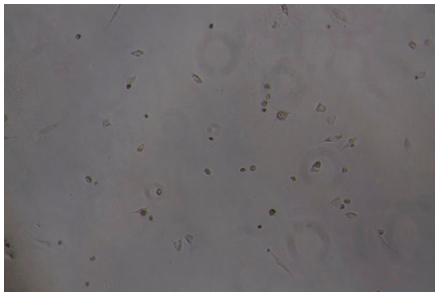
20×	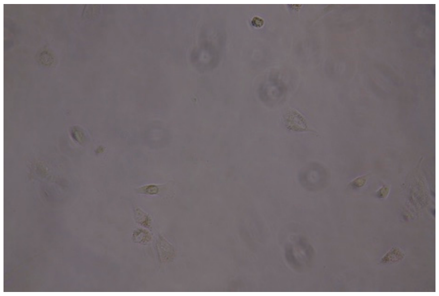	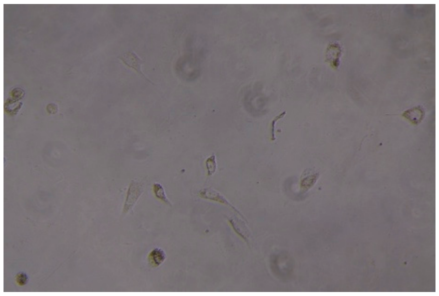
40×	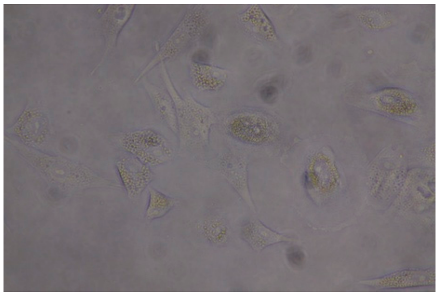	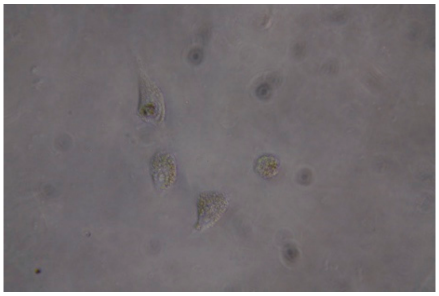
**7 days**		
4×	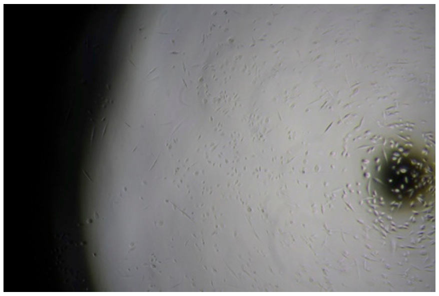	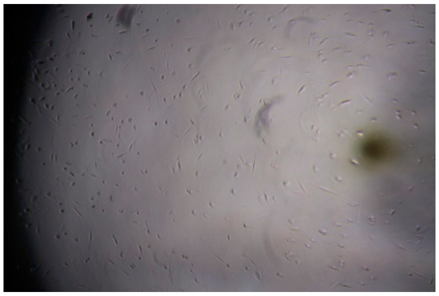

10×	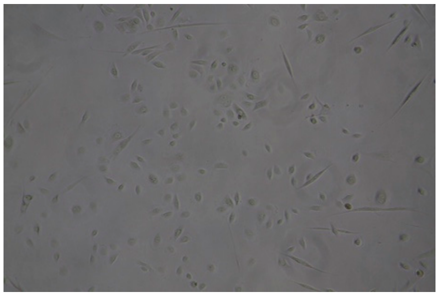	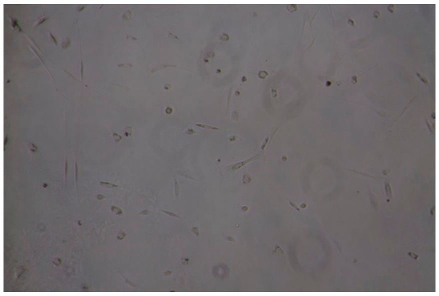
20×	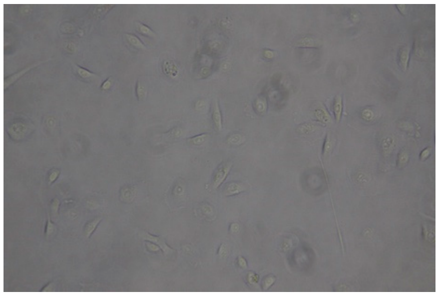	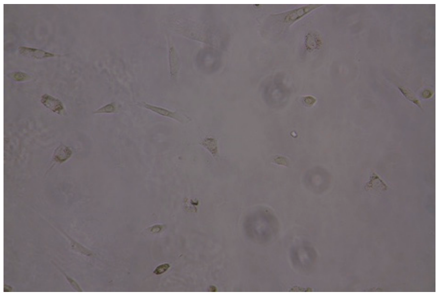
40×	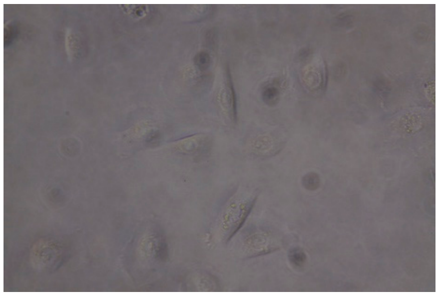	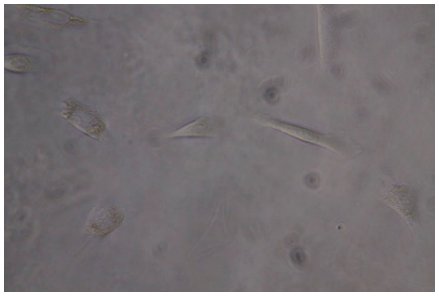
**10 days**		
4×	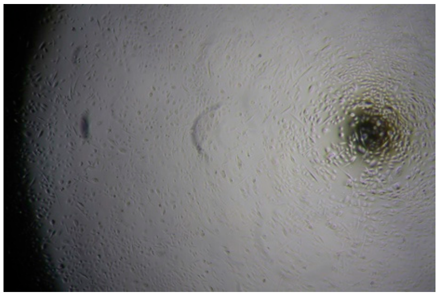	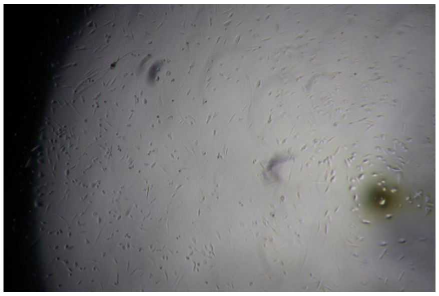
10×	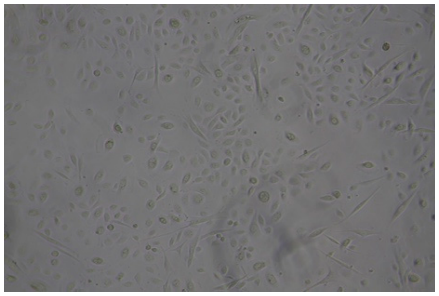	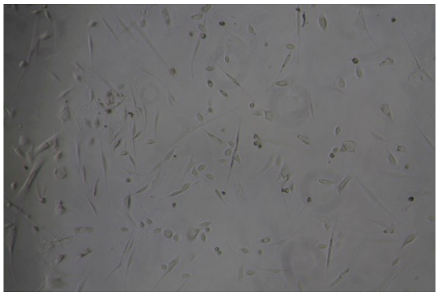

20×	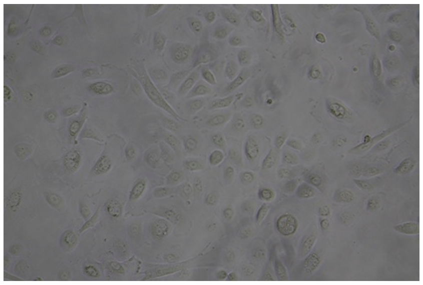	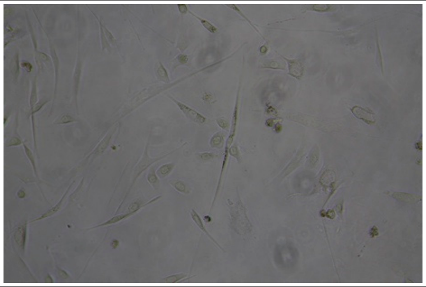
40×	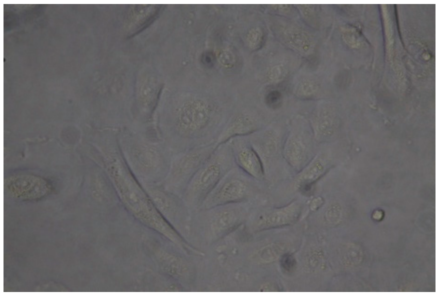	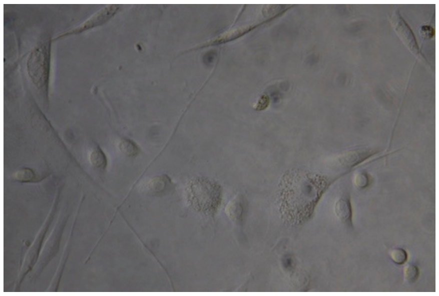
**14 days**		
4×	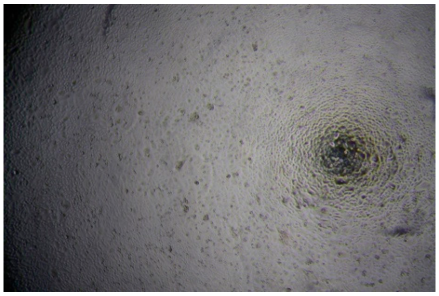	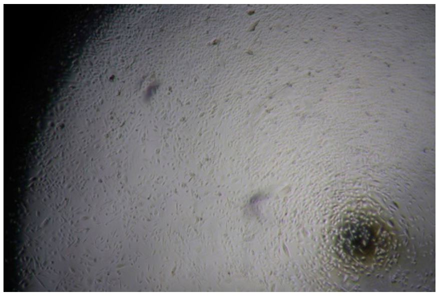
10×	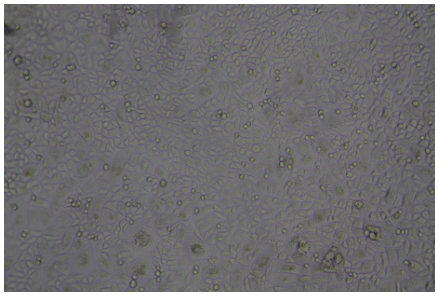	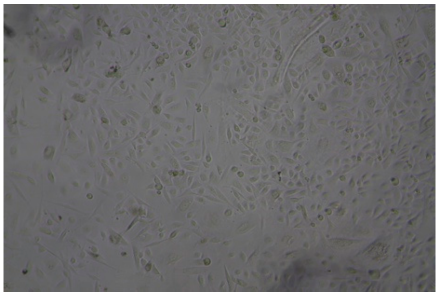
20×	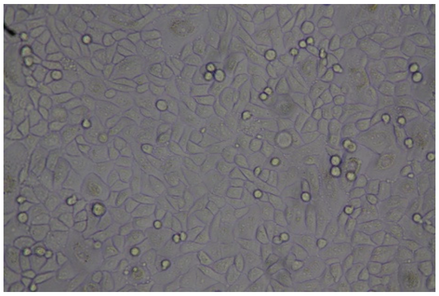	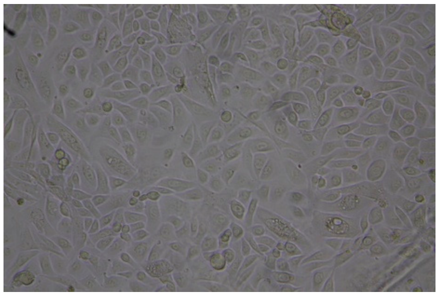

40×

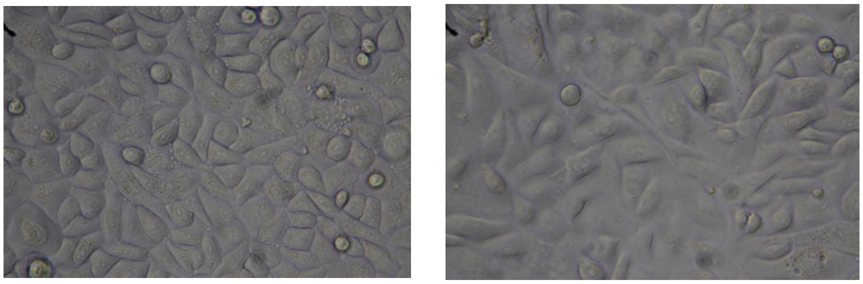

21 days

4×

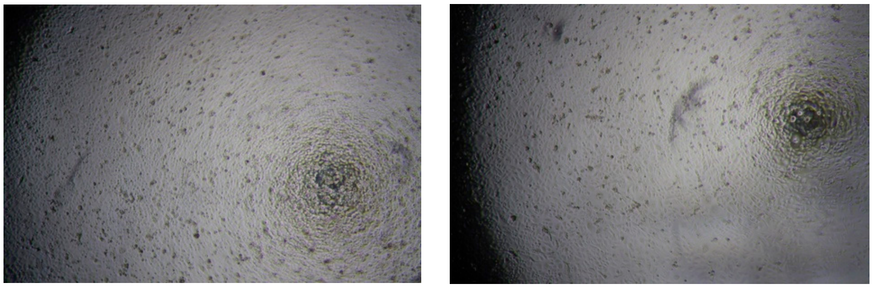

10×

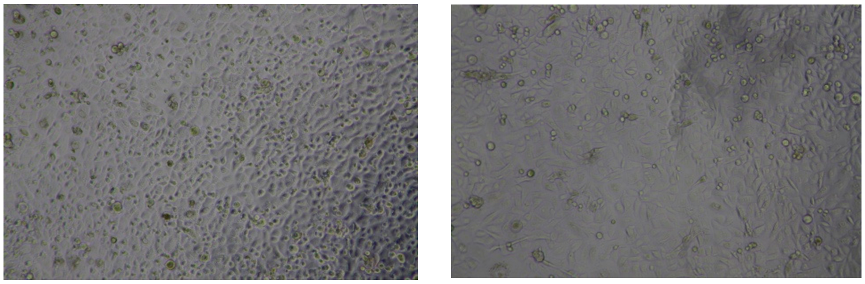

20×

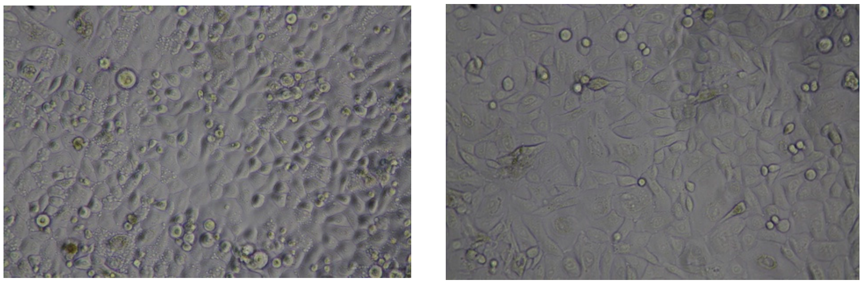

40×

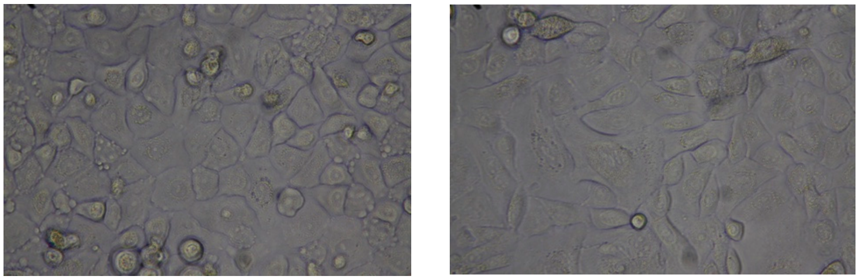

Figure S4. The light microscopic images (4×, 10×, 20× and 40× magnification) of human OMECs after 1, 3, 7, 10, 14 and 21 days of culture on HA scaffold dried for 3 days and coated with 1 mg/mL collagen IV. * Cultured human OMECs on coated plastic 24-well-plate with 1 mg/mL collagen IV.
